# Supplementary material for: Heterogeneous Macrophage Activation in Acute Skeletal Muscle Sterile Injury and mdx5cv Model of Muscular Dystrophy
Source: Int J Mol Sci. 2025 Aug 21;26(16):8098. doi: 10.3390/ijms26168098 (PMC12387070; doi:10.3390/ijms26168098)
Supplement: Supplementary file 1 [file ijms-26-08098-s001.zip › Supplementary information.pdf]

Supplementary Table S1. scRNA-seq Quality Control

**Sample ID**	***WT*_quadriceps**	***WT*_diaphragm**	***mdx^{5cv}*_quadriceps**	***mdx^{5cv}*_diaphragm**	**day 1_quadriceps**	**day 3_quadriceps**
**Estimated Number of Cells**	6,545	8,485	9,897	8,286	7,367	6,640
**Mean Reads per Cell**	43,703	33,343	30,252	34,137	35,440	44,464
**Median Genes per Cell**	962	1,746	1,800	1,761	2,257	2,926
**Median UMI Counts per Cell**	1,780	4,128	4,603	4,505	8,640	10,578
**Reads Mapped Confidently to Intronic Regions**	22.3%	17.5%	21.9%	20.7%	14.8%	16.4%
**Reads Mapped Confidently to Exonic Regions**	69.9%	74.4%	69.3%	71.1%	77.0%	74.0%
**Reads Mapped Confidently to Transcriptome**	68.3%	72.7%	67.8%	69.6%	74.6%	71.9%
**Q30 Bases in RNA Read**	94.3%	93.5%	94.4%	94.5%	93.9%	94.6%
**Fraction Reads in Cells**	85.5%	93.1%	94.3%	94.1%	96.9%	92.6%

Supplementary Table S2. Top 50 differentially expressed genes (DEGs) of different monocyte/macrophage sub-clusters

Cluster ID	Monocytes	IFNRMs	Pro-inflammatory	Gpnmb^{+}Spp1^{+}	MHCII^{hi}	TLF^{+}	Proliferating
Top 50 DEGs	Plac8	Rsad2	Cxcl3	Gpnmb	H2-Aa	Sepp1	Stmn1
	Hspa1b	Ifit3	Il1b	Fabp5	H2-Eb1	Retnla	Top2a
	Rgs1	Ifit1	Arg1	Spp1	Cd74	Lyve1	Pclaf
	Fosb	Ccl12	Cxcl1	Syngr1	H2-Ab1	Ccl8	Mki67
	mt-Nd4l	Isg15	Thbs1	Fabp4	H2-DMa	Folr2	Hmgb2
	Atf3	Cxcl10	Ppbp	Trem2	Tmem176b	Fcgrt	Birc5
	Ms4a4c	Ifit2	Clec4e	Lpl	H2-DMb1	Cd81	Tuba1b
	Ifi207	Cmpk2	Ptgs2	Hexb	Tmem176a	Shfm1	Pcna
	Ly6c2	Ifit3b	Fn1	Cd36	Pltp	Cd209f	Ube2c
	Apoc2	Slfn5	Cd14	Serpinb6a	Ly86	Cfh	Smc2
	Cxcr4	Ifi47	Ier3	Rgs1	Cd81	Trf	Tmpo
	Gsn	Irf7	Ccl2	Timp2	Mgl2	Mgl2	Rrm2
	Jmjd1c	Ccl8	Ccl6	Cd9	Trf	Serinc3	Mcm6
	Plec	Oasl2	Ccl7	Cd63	Axl	Selk	Mcm5
	BC005537	Phf11b	Chil3	Lipa	Clec4b1	45184	Smc4
	Hsph1	Ifi204	Hilpda	Vat1	Cybb	Xist	Cdk1
	Zfand5	Mndal	Slc7a11	Pld3	Slamf9	Rps27rt	Gmnn
	Spag9	Ifi203	Vcan	Gdf15	Hpgd	Tsc22d3	Lig1
	Osbpl8	Usp18	Ehd1	Slc27a1	Txnip	Cd163	Rrm1
	Ifrd1	Rtp4	Il1a	Sgk1	Cx3cr1	F13a1	Hist1h2ap
	Rtraf	Oasl1	S100a8	Folr2	Serinc3	Ltc4s	Dut
	Kcnn4	Ifi209	Tnfsf9	Cregl	Cxcl16	Cbr2	Tk1
	Emp1	Parp14	Slc16a3	Uap1l1	Mpeg1	2010107E04Rik	Ccna2
	Gadd45g	Stat1	Nlrp3	Itga6	C1qc	Fcna	Hist1h1b
	Pmepa1	Tor3a	Jdp2	Atp6v1a	Cd72	Gm9843	Atad2
	Aopep	Ifi211	Slfn2	Slc6a8	Ighm	Rpl23a-ps3	Cdca8
	Myh9	Phf11d	Ltb4r1	Msrb1	Lyz1	C1qc	Dek
	Ccr2	Ly6e	Pdpn	Tnfsf13	Ciita	C1qa	Mcm7
	Lmna	Trim30a	Phlda1	Hebp1	Rps27rt	Tceb2	Anp32b
	Msrb1	Mnda	Piml	Gyg	Klf2	Sepw1	Ranbp1
	Gng10	Bst2	Slpi	Il7r	AW112010	Gas6	Cks1b
	Ranbp2	Rnf213	Clec4d	Nceh1	Hspa1b	Minos1	Hist1h2ae
	Grcc10	Xaf1	Rab20	Lhfpl2	Ifngr1	Usmg5	Prc1
	Itgb5	Pnp	Hif1a	Gpr137b	Lpcat2	Gnb2l1	Cdca3
	Tmem119	Ms4a4c	Il1rn	Gusb	Tsc22d3	Fxyd2	Uhrf1
	Ckap4	Slfn1	Tnf	Anxa1	C1qb	Hpgd	Tpx2
	Itgav	Trafd1	Ifrd1	Fabp3	Ptprc	Rnaset2a	Mcm3
	Gas5	Sp100	Sdc4	Osbpl8	Mef2c	Clec10a	Tubb4b
	Tent5c	Zbp1	Mxd1	Cpd	Rassf4	Igfbp4	Tyms
	Itga6	Ddx58	Basp1	Lat2	Hspa1a	Wdr89	Nusap1
	Cct6a	Ifih1	Bcl2a1b	Scoc	Gm9843	Gm9493	Racgap1
	Tle5	Gbp2	Timp1	Tpp1	Rpl23a-ps3	Klf2	Nap1l1
	Runx3	Herc6	Prdx6	Smpdl3a	Tmcc1	Rps12-ps3	Ezh2
	Rasgef1b	Stat2	Socs3	Fcrls	Man2b1	Ccl24	Hmgn1
	Gm34084	Fgl2	Gadd45b	Soat1	Retnla	Rpl6l	Nucks1
	Resf1	Samhd1	F10	Pmp22	Gpr34	Rgs10	Plp2
	Chka	Samd9l	Odc1	C3ar1	Clec4a3	Fcrls	Dnmt1
	Slc38a2	Ifi27l2a	Osm	Tnfsf12	Pld4	Igf1	Pbk
	Emilin2	Ifi213	Cdkn1a	Esyt1	C1qa	AI607873	Incenp
	Lsp1	Oas3	Kdm6b	Sptssa	Lair1	Fam46a	Cenpf

Supplementary Table S3. Functional enrichment analysis of different monocyte/macrophage sub-clusters

**Monocytes**	phagocytosis
	cell adhesion
	integrin-mediated signaling pathway
	cell extravasation
	reactive oxygen species metabolic process
	cellular detoxification
	type I interferon signaling pathway
**IFNRMs**	Type I/II IFN signaling
	Endocytosis/Antigen processing and presentation
	RIG-I/MDA-5 signaling pathway
	NOD-like receptor signaling pathway
	Cytosolic DNA-sensing pathway
	IL-10 anti-inflammatory signaling pathway
**Pro-inflammatory**	acute inflammatory responses: 1) TLR/TNFa/NF-kB signaling; 2) Myeloid leukocytes chemotaxis; 3) Myeloid leukocytes activation; 4) C-type lectin signaling; 5) inflammasome; 6) phagosome.
	stress responses: 1) ROS/RNS metabolism process; 2) HIF signaling
	VEGFA-VEGFR2 signaling
	Apoptosis
**Gpnmb^{+}Spp1^{+}**	lipid metabolism, transport, and storage
	Plasma lipoprotein assembly, remodeling, and clearance
	PPAR signaling pathway
	Lysosome
	Phagosome
	Glycosaminoglycan degradation
	Energy metabolism: 1) ATPase activity; 2) protein catabolic process;
	Antigen processing-Cross presentation
**MHCII^{hi}**	Antigent processing and presentation via MHC class II
	Th1 and Th2 cell differentiation
	Complement activation classical pathway
	regulation of cell motility
**TLF^{+}**	Lipid metabolis
	Lysosome
	Efferocytosis
	integrin complex
**Proliferating**	Cell cycling

Supplementary Table S4. Cell number and percentage of each cluster to total monocytes/macrophages

Sample ID	Quadriceps_*WT*		Diaphragm_*WT*		Quadriceps_day 1		Quadriceps_day 3		Quadriceps_*mdx*^{5cv}		Diaphragm_*mdx*^{5cv}	
	Number	Percentage	Number	Percentage	Number	Percentage	Number	Percentage	Number	Percentage	Number	Percentage
**Monocytes**	3	0.4348	0	0	90	9.375	605	45.7294	29	5.6974	72	17.9551
**IFNRMs**	21	3.0435	7	2.1084	0	0	38	2.8723	10	1.9646	15	3.7406
**Pro-inflammatory**	0	0	0	0	839	87.3958	31	2.3432	0	0	1	0.2494
**Gpnmb$^+$Spp1$^+$**	1	0.1449	3	0.9036	4	0.4167	380	28.7226	98	19.2534	71	17.7057
**TLF$^+$**	446	64.6377	161	48.4940	24	2.5	7	0.5291	9	1.7682	49	12.2195
**MHCII^{hi}**	186	26.9565	158	47.5904	1	0.1042	6	0.4535	353	69.3517	187	46.6334
**Proliferating**	33	4.7826	3	0.9036	2	0.2083	256	19.3500	10	1.9646	6	1.4963
**Total**	690	100	332	100	960	100	1323	100	509	100	401	100

Supplementary Table S5. Top 50 differentially expressed genes (DEGs) of different monocyte/macrophage sub-clusters

Cluster ID	Gpx3$^+$	MHCII^{hi}	Ccl8$^+$	Spp1$^+$Arg1$^+$	Gpnmb$^+$Spp1$^+$	Pro-inflammatory	Monocytes	MHCII^{hi}	IFNRMs
Top 50 DEGs	Ldhb	H2-Eb1	Ccl8	Spp1	Gpnmb	Gja1	Vcan	Clec4b1	Ifit1bl1
	Tspan13	H2-Aa	Cbr2	Fabp4	Atp6v0d2	Ccl7	Slpi	H2-Eb1	Cmpk2
	Ramp1	H2-Ab1	Clec10a	Htr2b	Syngr1	Ccl2	Hp	H2-Ab1	Ifit3b
	Ypel3	Lag3	Stmn1	Arg1	Stra6l	Pf4	Dmkn	Tnip3	Ifit1
	Gpx3	Cd74	Igfbp4	Dhrs9	Fabp5	Ccl6	Trem1	H2-Aa	Rsad2
	Pink1	Myl1	Fxyd2	F7	Gm1673	Gar1	F10	Cd74	Ifit2
	Cx3cr1	Ciita	Gas6	Zranb3	Fbxo32	Mrps28	Ifitm6	Olfml1	Tnfsf10
	Rgs2	Acta1	Folr2	Cgnl1	Ldhb	Utp20	Ptgs2	Plbd1	Serpina3g
	Bckdha	Mylpf	Selenbp1	Rnf128	Galns	Fn1	Plcb1	H2-DMb1	Ifit3
	Ppfia4	Cd81	Fcgrt	Pdpn	Naglu	Odc1	Tarm1	Rnd3	Mx1
	Cyp27a1	H2-DMb1	Ctla2b	Cd36	Itgax	Akr1b8	Mcemp1	Lsp1	Iigp1
	Olfml3	Tmem176a	Ltc4s	Jag1	Mgst1	Timp1	Cxcl3	Hspa1b	Tgtp2
	Il16	Vcam1	Smagp	Chst11	Pld3	Slc39a14	Chil3	Naaa	Cxcl10
	Pltp	Tmem176b	Cfh	Cdk20	Msrb1	Rai14	Ltb4r1	Dnajb1	Gbp2
	Il18bp	Batf3	Npl	Pde4d	F7	Slc35e4	Met	Stap1	Gbp5
	Tnfaip8l2	Clec4b1	Hgsnat	Fabp5	Anpep	Eef1e1	Uck2	Ccr2	Usp18
	Lpcat2	Cd72	Col1a2	Slc6a8	Gpr137b	Pdpn	Slc7a11	Hsph1	Ifi47
	Gyg	Hes1	Stard8	Rgcc	Nr1h3	Srm	S100a8	Traf1	Trim30c
	Rgs1	Hpgd	Mrc1	Adam8	Hebp1	Dok2	Slc16a3	Gpr183	Isg15
	Abi3	Hspa1b	Pmp22	Igf2r	Arl11	Oaf	Agpat9	Slamf9	Herc6
	Man2b1	Egr1	Cela1	Bcar3	Il18bp	Mettl1	Mmp19	H2-DMa	Phf11a
	Mxd4	Scimp	Abca9	Fam20c	Gngt2	Jag1	Il1a	Cd72	Gm4955
	Oxa1l	H2-DMa	Fcrls	Papss2	Creg1	Ifi202b	Cd177	Dcn	Isg20
	Ssh2	Hspa1a	Ap2a2	Nav2	Smpdl3a	Ccnd1	Gsr	Etv3	Igtp
	Tnrc6b	Slamf9	Sbf2	Cd109	Echs1	Kcnn4	Thbs1	Cytip	Gbp7
	Hscb	Axl	Ednrb	Fabp3	Dpp7	Tubb6	Igfbp6	Bcl2a1d	Irgm2
	March1	Gpr34	Rab11fip5	Fn1	Atp1a3	Phldb1	Il1b	Gpr132	Slfn1
	Cir1	Slamf8	Fh1	Adssl1	Vegfb	Olr1	Clec4e	Ptger4	Pydc4
	Tmem86a	Fam26f	Ddx60	Mmp8	Gsto1	Rrp15	Adora2a	Samsn1	Oasl1
	Neurl3	H2-Q7	Sgpp1	Rai14	Socs6	Abce1	Irg1	H2-Q7	Trim21
	Klhl6	Ppfia4	F13a1	Cd24a	Ahnak2	Ddx39	Klra2	Hspa1a	Ifi44
	Pnpla2	Dnajb1	Rnasel	Slc27a1	Cd9	Marcksl1	Sgms2	Pmaip1	Stat2
	Bmyc	Hsph1	Cd33	Arhgap24	Abi3	Tubb4b	B3gnt5	Nr4a3	Phf11b
	Atp13a2	Itgax	Maf	Cadm1	Stmn1	Manf	Flrt3	Mylpf	Phf11d
	Limd2	Pmaip1	Serpinb6a	Mctp1	Bhlhe41	Nomo1	Ccr1	Tmem176a	Gm5431
	Cpq	Pou2f2	Fchsd2	Il7r	Timp2	S100a10	Ly6c2	Fam129a	Gbp3
	Hfe	Tmcc3	Stab1	Prmt2	Tecpr1	Il10	Clcf1	Arl5c	Pyhin1
	Mylip	Slco2b1	Lrp6	Slc41a2	Pla2g16	Mdn1	Timp1	St8sia4	Treml2
	Tifab	Cpq	Reps2	Pf4	Lyst	Gda	Rab11fip1	Tmem176b	Themis2
	Trf	Dcn	H2afv	Myo1e	Hexb	S100a4	Plac8	Axl	Oas3
	Fos	Rhoh	Dab2	Inf2	Comtd1	Tuba1c	Ccl6	Maff	Slc25a22
	Gramd1b	Gdf15	Fbxw4	Ampd3	AU020206	Cbr2	Cxcl2	Napsa	Tor3a
	Fam105a	Btg2	Tbc1d14	Vat1	Hpse	Slc7a8	Gda	Itgax	Gbp9
	Tep1	Txnip	Prune2	Anxa1	Paox	Creld2	Ccl9	Dusp2	Il15
	Pla2g15	Spic	Slc9a9	Akr1b8	Pon3	Tfrc	Fdps	Adrbk2	Tpst1
	AB12461	Trf	Siglec1	Atf1	Gyg	Mdm2	Itgb7	Cd83	Nt5c3
	Adipor1	Lpcat2	Slco2b1	Hk3	Spp1	Ccl9	Slc39a14	Sema4d	Irgm1
	Ccni	Klf2	Cryl1	Tmem120a	Gstm1	Plec	Slc2a1	Sowahc	Ddx60
	Arhgap15	Tsc22d3	Hfe	Psmd1	Bnip3l	Nop58	Clec4d	Sparc	Ccl12
	Irf8	Il16	Ppp1cc	Tmem8	Plxnc1	Lmna	C3	Id3	
